# Epithelial–mesenchymal transition: an organizing principle of mammalian regeneration

**DOI:** 10.3389/fcell.2023.1101480

**Published:** 2023-10-26

**Authors:** Kamila Bedelbaeva, Benjamin Cameron, John Latella, Azamat Aslanukov, Dmitri Gourevitch, Ramana Davuluri, Ellen Heber-Katz

**Affiliations:** ^1^ Lankenau Institute for Medical Research (LIMR), Wynnewood, PA, United States; ^2^ The Wistar Institute, Philadelphia, PA, United States

**Keywords:** MRL and C57BL/6 mice, chromatin remodeling, EMT, G2 cell cycle arrest, EVI-5, EZH2, HIF-1α, blastema

## Abstract

**Introduction:** The MRL mouse strain is one of the few examples of a mammal capable of healing appendage wounds by regeneration, a process that begins with the formation of a blastema, a structure containing de-differentiating mesenchymal cells. HIF-1α expression in the nascent MRL wound site blastema is one of the earliest identified events and is sufficient to initiate the complete regenerative program. However, HIF-1α regulates many cellular processes modulating the expression of hundreds of genes. A later signal event is the absence of a functional G1 checkpoint, leading to G2 cell cycle arrest with increased cellular DNA but little cell division observed in the blastema. This lack of mitosis in MRL blastema cells is also a hallmark of regeneration in classical invertebrate and vertebrate regenerators such as planaria, hydra, and newt.

**Results and discussion:** Here, we explore the cellular events occurring between HIF-1α upregulation and its regulation of the genes involved in G2 arrest (*EVI-5*, *γH3*, *Wnt5a*, and *ROR2*), and identify epithelial–mesenchymal transition (EMT) (Twist and Slug) and chromatin remodeling (EZH-2 and H3K27me3) as key intermediary processes. The locus of these cellular events is highly regionalized within the blastema, occurring in the same cells as determined by double staining by immunohistochemistry and FACS analysis, and appears as EMT and chromatin remodeling, followed by G2 arrest determined by kinetic expression studies.

## Introduction

It is well-accepted that mammals do not regenerate appendages, whereas amphibians have an impressive regenerative ability (Stocum, 1984; Gardiner and Bryant, 1996; Brockes and Kumar, 2005). In addition to amphibians, extensive regeneration is also observed in planaria, starfish, sea cucumbers, and hydra (Sanchez Alvarado, 2006; Ramirrez-Gomez and Garcia-Arraras, 2010; Galliot, 2012).

A structural key to the regenerative process is the early formation of the blastema, which develops after a wound. The amphibian accumulation blastema found in the re-growing limb is a mass of multipotent cells with stem cell markers, believed to be derived from cells migrating into the wound site or from local cells de-differentiating after the process of re-epithelialization and closure of the limb amputation wound. The wound epidermis, with the formation of an apical ectodermal cap (AEC) (Stocum, 2017), comprises active epithelial cells producing factors (Christensen and Tassava, 2000) such as fibronectin and matrix metalloproteinases (MMPs) (Campbell et al., 2011; Godwin et al., 2013). These factors show potential to affect communication with the mesenchyme, and for this to occur, the basement membrane (BM) must be broken down (Vinarsky et al., 2005). Once that happens, mesenchymal cells begin to accumulate in the space above the cartilage and under AEC. However, interestingly, little mitosis is observed in the blastema (Tassava et al., 1974).

This lack of mitosis has been a point of interest for the regeneration community for some time. It was shown that blastema cells in the amphibian (axolotls and salamanders) had a high level of DNA synthesis with continuous labeling (approximately 80%) but a very low level of mitosis (approximately 0.4%) (Stocum and Dearlove, 1972; Tassava et al., 1974; Mescher and Tassava, 1976; Stocum and Crawford, 1987). A reduction in blastemal mitosis was observed not only in amphibians (Tassava et al., 1974) but also in hydra (Park et al., 1970; Buzgariu et al., 2018), which have regenerative cells which go through cell cycle entry and then stop in G2/M, and planaria (Salo and Baguna, 1984; Sahu et al., 2021). In the mammalian liver, which is known to regenerate, G2 arrest is observed in adult hepatocytes which are 70% tetraploid (Michalopoulos, 2013; Zhang et al., 2019).

*In vitro* studies with newt myotubes showed serum stimulation, cell cycle entry, and then, G2 arrest (Tanaka et al., 1997). This mitotic reduction and G2M arrest have features similar to those found in the regenerative MRL blastema, and in both normal and blastemal cells in culture but not in non-regenerative C57BL/6 cells (Bedelbaeva et al., 2010; Heber-Katz et al., 2013). Cell cycle analysis revealed that the majority of these cultured cells were found to be in G2M, showed a DNA damage response expressing p53 and γH2AX, and lacked the expression of the p21^cip/waf^ protein (CDKN1a), a key G1 cell cycle checkpoint regulator. The lack of p21 in the regenerating MRL mouse predicted that its elimination in otherwise non-regenerating mice would convert these to regenerators. Indeed, p21 KO mouse ear holes could regenerate (close ear holes) like MRL (Bedelbaeva et al., 2010; Heber-Katz et al., 2013).

An obvious question is why or what these cells do during G2 arrest without mitosis. Our aim was to identify such cells in the regenerating MRL mouse ear tissue and examine molecules that might reveal information about this.

We used markers of G2 arrest (EVI-5, γH3, wnt5a, and ROR2) to identify such cells. We show here that these cells appear beneath the day-7 MRL wound epidermis and in the newly forming blastema but not in B6 tissue. Interestingly, these cells were located in a region where epithelial–mesenchymal transition (EMT) seemed to occur (a region where basement membrane breakdown occurred). Using the EMT markers (Twist and Slug), we found a significant overlap between the two populations.

The breakdown of BM between the epidermis and dermis is also a hallmark of amphibian regeneration and, when blocked in the axolotl, leads to acute scar formation and a complete cessation of the regenerative response (Stocum and Dearlove, 1972; Stocum and Crawford, 1987). Similar basement membrane breakdown is observed in the MRL mouse ear hole (Gourevitch et al., 2003) but not in the C57BL/6 scarring response. Major molecules involved in this BM remodeling process include MMPs (Godwin et al., 2013) in both amphibians and the MRL mouse (Gourevitch et al., 2003). Basement membrane loss has been associated with an EMT response (Spaderna et al., 2006a).

One possible function of such cells resting in G2/M was DNA repair or chromatin remodeling. Thus, we used markers for chromatin remodeling (EZH2 and H3K27me3) and found that again, there was a significant overlap. Since tissue labeling is not always exact, we isolated day-7 blastemal cells from the MRL mouse ear blastema and showed using FACS analysis that over half of the cells showed triple labeling.

Finally, we previously showed that the hypoxia-inducible factor or HIF-1α is highly expressed in the day-7 MRL blastema (Clark et al., 1998; Zhang Y. et al., 2015). Here, we show that HIF-1α is also a central activator of EMT and chromatin remodeling.

## Materials and methods

### Animals

MRL/MpJ female mice were obtained from The Jackson Laboratory; C57BL/6 female mice were obtained from Taconic Laboratories. Mice were used at approximately 8–10 weeks of age in all experiments under standard conditions at the LIMR and the Wistar Institute Animal Facilities. The mice were ear-punched and euthanized on days 2, 3, 5, and 7, and ear pinnae were removed, as indicated and as previously described (Clark et al., 1998).

### Tissue preparation, immunohistochemistry, and microscopy

Tissue from hole-punched ears was fixed with Prefer fixative (the active ingredient is glyoxal) (Anatech) overnight, washed in H_2_0, and placed in 70% ETOH. The tissue was embedded in paraffin and then cut into 5-μm-thick sections. Before staining, slides were dewaxed in xylene and rehydrated. Antigen retrieval was performed by autoclaving for 20 min in 10 mM sodium citrate, pH 6.0. The tissue sections were then treated with 0.1% Triton, and nonspecific binding was blocked with 4% BSA (A7906; Sigma) for 1 h. The primary antibodies and matched secondary antibodies used for immunohistochemistry (IHC) are shown in Table 1.

**TABLE 1:** Primary and secondary antibodies used for IHC.

	Primary antibody			Secondary antibody			
	Company	Cat. no.	Dilution	All from molecular probe	Company	Cat. no.	Dilution
HIF-lα	Abcam	ab2185	1:1,000	Alexa Fluor 488 goat anti-rabbit IgG	Molecular Probe	A11008	1:200
EV15	Millipore	ABN194	1:100	Alexa Fluor 568 goat anti-rabbit IgG	Molecular Probe	A11036	1:300
γH3	Upstate	06-570	1:100	Alexa Fluor 568 goat anti-rabbit IgG	Molecular Probe	A11036	1:300
Wnt5a	R&D	BAF645	1:150	Alexa Fluor 568 goat anti-rabbit IgG	Molecular Probe	A11005	1:200
ROR2	Cell Signaling	4,105	1:100	Alexa Fluor 568 goat anti-rabbit IgG	Molecular Probe	A11036	1:200
Twist	Santa Cruz	Sc81417	1:150	Alexa Fluor 488 goat anti-rabbit IgG	Molecular Probe	A21121	1:200
BMI-1	Santa Cruz	Sc390443	1:250	Alexa Fluor 488 goat anti-rabbit IgG	Molecular Probe	A21121	1:200
H3k27me3	Abcam	Mab6002	1:100	Alexa Fluor 488 goat anti-rabbit IgG	Molecular Probe	A21121	1:200
EZH2	Thermo Scientific	MM-15101	1:100	Alexa Fluor 488 goat anti-rabbit IgG	Molecular Probe	A21121	1:200
EZH2	Invitrogen	3210608	1:100	Alexa Fluor 488 goat anti-rabbit IgG	Molecular Probe	A11008	1:200

For histological stains, tissue sections were treated the same, as explained in the previous section, and then stained with hematoxylin (Leica Microsystems, # 3801562) and eosin (Leica Microsystems, #3801602). For IHC, tissue sections were then treated with 0.1% Triton, and nonspecific binding was blocked with 4% BSA (A7906; Sigma) for 1 h. The primary antibodies and matched secondary antibodies used for IHC are shown in Table 1. The slides were washed, rehydrated, cleared with xylene, and coverslipped with Permount mounting medium (Fisher, SP15-500). Staining was visualized for fluorescent labeling using a fluorescent Olympus (AX70) microscope and a DP74 camera and cellSens software for image analysis, or a bright-field microscope for H&E staining, as previously described (Zhang Y. et al., 2015).

For cultured cell staining, primary fibroblast-like cell lines from ear tissue were established from MRL and B6 female mice and then grown in DMEM–10% FBS supplemented with 2 mM L-glutamine and 100 IU/mL penicillin–streptomycin, and maintained at 37°C, 5% CO_2_, and 21% O_2_. For immunohistochemical staining, fibroblasts were grown on coverslips in DMEM with 10% FBS at 37°C in a humidified 5% CO_2_ incubator. The coverslips were rinsed with 1× PBS; the cells were fixed in cold methanol (−20°C) for 10 min, rinsed with 1× PBS, treated with 0.1% Triton-X100, and then incubated with the appropriate primary and secondary antibodies (Table 1). Photomicrographs were produced using the fluorescent microscope (Olympus AX70) and a DP74 camera, with cellSens Standard software for image analysis.

Confocal images were captured using a Leica TCS SP5 II laser scanning confocal microscope (Leica Microsystems, Inc., Deerfield, IL) using AOBS and sequential scanning with 405, 488, and 561-nm laser lines. Individual frames and short z-stacks were acquired at maximum resolution with a 63 × 1.4 NA objective, following Nyquist criteria. Post-processing for maximum projection and noise reduction was carried out using Leica LAS-AF software and exported to .tif files.

### FACS analysis

Day-7 MRL ear donuts from 2-mm ear punches were generated using a 4-mm punch to retrieve the tissue of interest. These donuts were teased apart, treated with dispase and collagenase, and then immediately stained with multiple antibodies without culturing. Cells were fixed in 4% paraformaldehyde for 15 min at room temperature and then washed in excess PBS. Permeabilization was achieved by adding ice-cold methanol to pre-chilled cells, while gently vortexing to a concentration of 90%, and then placing the cells on ice for 10 min. After washing in excess PBS, the cells were stained in 100 µl of staining buffer (PBS +2% FBS) containing PE Mouse SNAI2/Slug (BD Biosciences 564615), EZH2 eFluor 660 (Thermo Fisher 50-9867-82), and Tri-Methyl-Histone H3 (Lys27) PE-Cy7 (Cell Signaling 91611S) (Table 2), and incubated for 30 min at 4°C protected from light. Cells were resuspended in PBS, and the fluorescent signals were acquired using the BD FACS Canto II system. Compensation was performed using UltraComp eBeads (Thermo Fisher 01-2222-41). Cell gates were drawn based on FSC/SSC, and doublets were discriminated prior to analysis. Percentages were obtained using FlowJo software.

**TABLE 2:** Directly labeled antibodies used for FACS analysis.

Specificity	Label	Company	Cat. #
Slug/SNAI2	PE	BD Biosciences	564615
EZH2	eFlour660	Thermo Fisher	50-9867-82
H3K27me3	PE-Cy7	Cell Signaling	91611S

## Results

### Analysis of cells in G2M using multiple cellular markers

**FIGURE 1:**
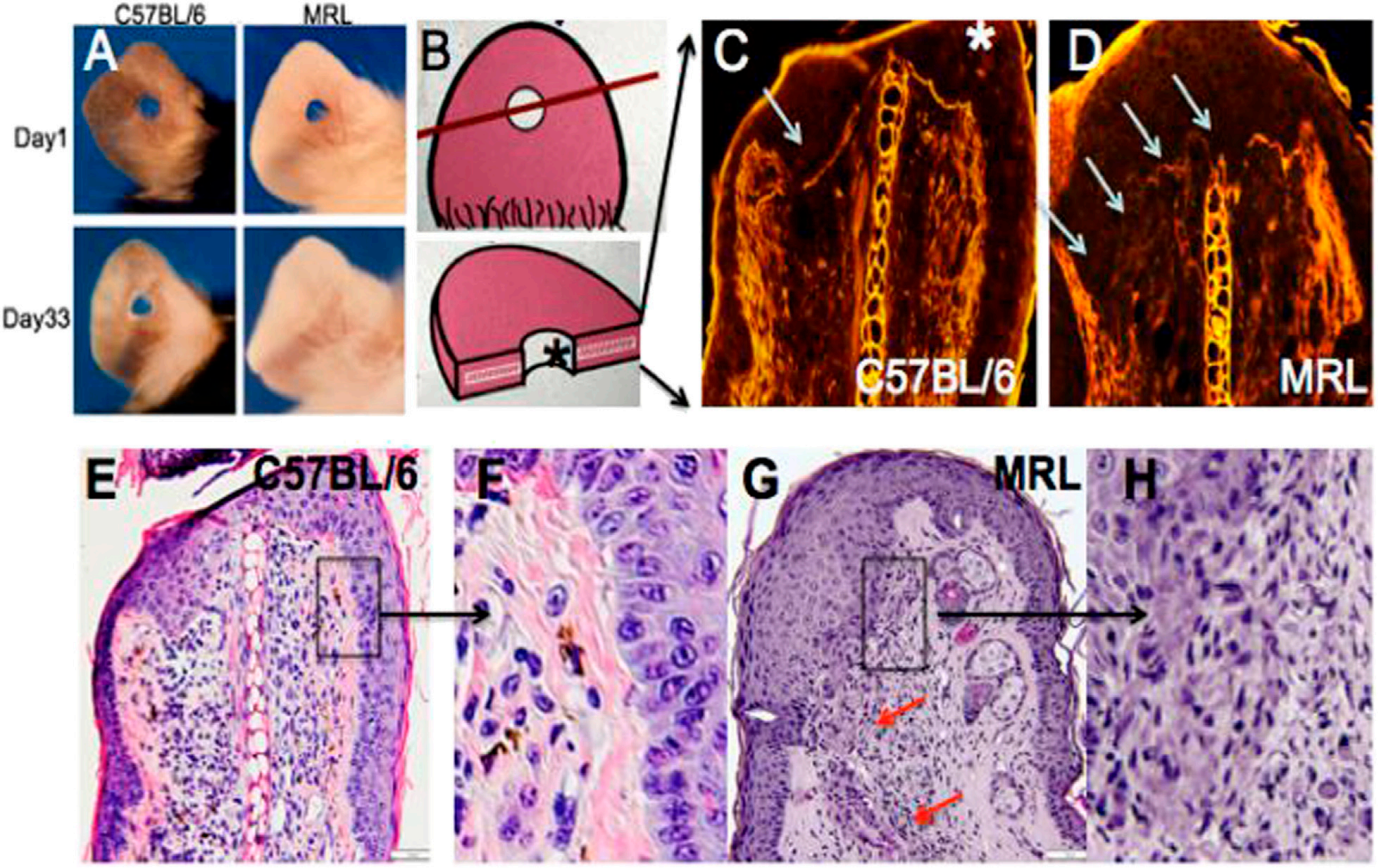
Ear hole closure, basement membrane breakdown, and differences in epithelial–mesenchymal borders on day 7 post-ear punching. The MRL mouse when ear-punched with a 2.1-mm punch shows complete ear hole closure **(A)** unlike any other mouse strain such as C57BL/6 or B6 (Clark et al., 1998). The processes have been identified to be similar to amphibian regeneration. A diagram of how the ear hole is cut is shown in **(B)**. The upper panel shows the ear pinna with the hole and a line showing how the ear was cut. The following panel shows the ear section with the asterisk indicating the top of the section. One of the early events in both amphibian and MRL regeneration is the breakdown of MRL but not the B6 basement membrane seen in **(C,D)** (white arrows). Here, injured tissue is stained with H&E, and epifluorescence shows stained protein levels, and the white line between the epidermis is seen in B6 but absent in MRL. Examination of the organization of the epidermal/dermal boundary after H&E staining shows a discrete border with a clear and organized basal epidermis in B6, with the boxed area **(E)** magnified ×3 in **(F)**, unlike that seen in the MRL boxed in area **(G)** and magnified ×2 in **(H)** with a disorganized and irregular border and no basal epidermis, also seen in other areas of the ear (red arrows). **(A)** is reproduced from Clark et al. (1998). **(E)** shows a measuring bar = 50 microns and applies to **(C,D,E,G)**.

For the analysis, 2.1-mm holes were created in the ear pinnae of female MRL and C57BL/6 mice. By day 33, the MRL ear hole wounds completely closed with lack of scarring, while the B6 ear holes remained for life with scarring along the hole perimeter [(Clark et al., 1998), Figure 1A)]. Early in the MRL regenerative response (day 7), the basement membrane disappeared in MRL under the wound epidermis but not in the non-regenerative B6 (Figures 1C, D, arrows). H&E sections show the distinct border between the B6 epidermis and dermis (Figures 1E, F), but the lack of cellular organization between the dermis and epidermis in the MRL wound site (red arrows, Figures 1G, H) suggests an ongoing EMT response.

**FIGURE 2:**
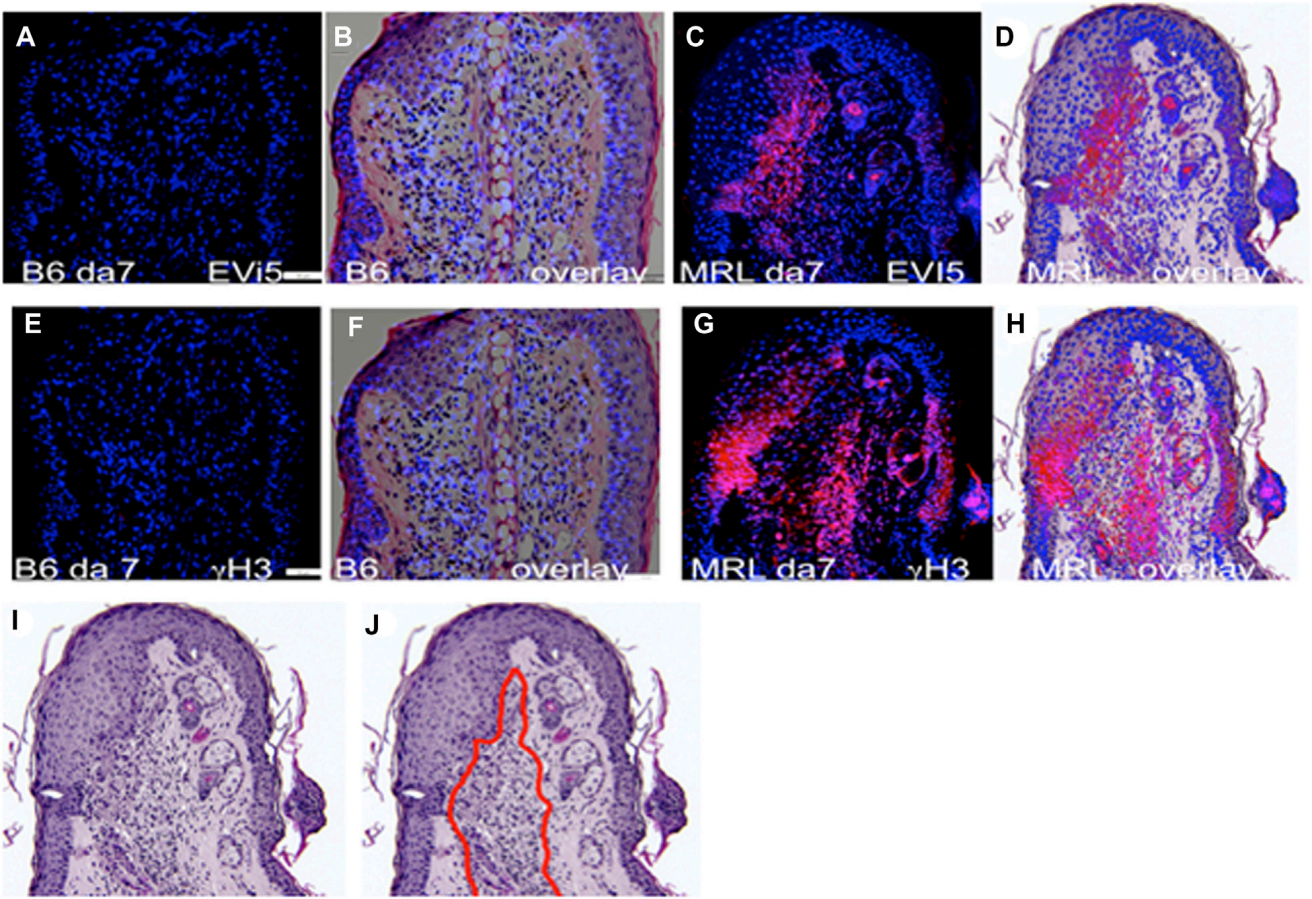
Ear tissue stained with G2M markers. B6 and MRL ear tissues from day 7 post-injury are stained with an antibody to EVI5 **(A–D)**, γH3 **(E–H)**, and DAPI **(A–H)**. B6 and MRL stained tissue is overlaid on a consecutive H&E-stained section from the same block. Staining of Wnt5a (Supplementary Figures S1C, D) and the Wnt5a receptor ROR2 (Supplementary Figures S1E, F) was also carried out. The zone of reactivity is based on the common staining region and cellular changes seen next to the regenerating epidermis in the MRL H&E section **(I),** which we called the “EMT zone” (surrounded by a red line) **(J)**. Two blocks with ear holes from two mice/strains and three consecutive sections per slide were stained per antibody. Data from one block for each strain are shown. In **(A,E)**, the measuring bar = 50 microns but applies to all figures.

Given the past known deficit in mitotic activity in the blastema, along with the finding that cells from the MRL blastema show an unusually high level of G2M arrest (Supplementary Figures S1A, B), we questioned where such cells in the healing ear tissue might be found in order to study them further. Consecutive serial sections collected from paraffin-fixed, hole-punched MRL and C57BL/6 mouse ears 7 days after injury were stained for G2M arrest markers, including EVI-5 and phospho-H3 (γH3) (Figures 2A–H), as well as wnt5a and ROR2, which were also considered G2M markers (Supplementary Figures S1C–F). These molecules were previously shown to have increased expression in the MRL ear compared to the B6 ear (Heber-Katz et al., 2013; Zhang Y. et al., 2015). B6 tissue showed little or no IHC staining for any of the G2M markers, compared to that observed in MRL tissue. MRL and B6 IHC images were overlapped onto H&E images (Figures 2B, D, F, H) from a consecutive slide. MRL IHC for these four molecules showed similar but not identical localization, possibly due to differences in the level of the ear wound embedded in the paraffin block. However, a general finding was that staining was observed in the area of the epithelial–mesenchymal margin (Supplementary Figures S1D, F; Figures 2D, H). This staining pattern suggested that we might see co-expression of G2 markers with an EMT marker, which was carried out by co-staining the same slide for multiple markers.

### Co-expression of G2M and EMT markers in the same region of the injured ear and in individual cells found there

**FIGURE 3:**
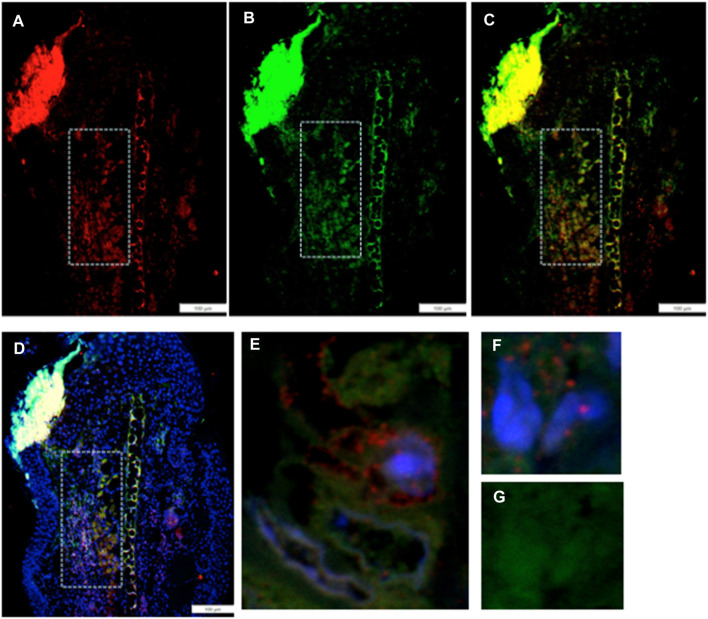
Ear tissue double-stained with the G2M marker EVI-5 and the EMT marker Twist1. **(A–D)** show a comparison of the same region in the MRL ear tissue (white boxes) from day 7 post-injury stained with an antibody to **(A)** EVI5 (red), **(B)** Twist1 (green), **(C)** a+b, and **(D)** a+b + DAPI (blue) and found in the “EMT zone.” Confocal images are seen in **(E–G),** showing co-staining in both the nucleus and cytoplasm of individual cells. EVI-5 is known to stain in the nucleus, nuclear membrane, and cytoplasm associated with tubulin and the cytoskeleton. Two blocks with ear holes from two different MRL mice and three consecutive sections per slide were double-stained. Data from one block are shown. DAPI staining shows the nuclei. The measuring bar = 100 microns. Note: regions of intense staining in the upper left corner of **(A–D)** are due to folded tissue in the slide preparation and are an artifact.

IHC co-staining of day-7 ear tissue was carried out with both the G2 marker EVI-5 and EMT marker Twist, as observed in Figure 3. The locus of Twist1 (green) staining in the day-7 MRL ear was observed in select regions (Figures 3B, C). In those Twist1-positive regions, EVI-5 (red) showed a similar pattern of staining (Figures 3A, C). High-magnification confocal images show co-staining of individual cells for both markers, with EVI-5 in and around the nuclear membrane and in the cytoplasm, and Twist1 staining more diffusely in both the nucleus (DAPI + nuclei, blue) and cytoplasm (Figures 3E–G). These cells were observed throughout the co-stained ear regions.

### Co-expression of EMT and chromatin remodeling markers in individual cells

**FIGURE 4:**
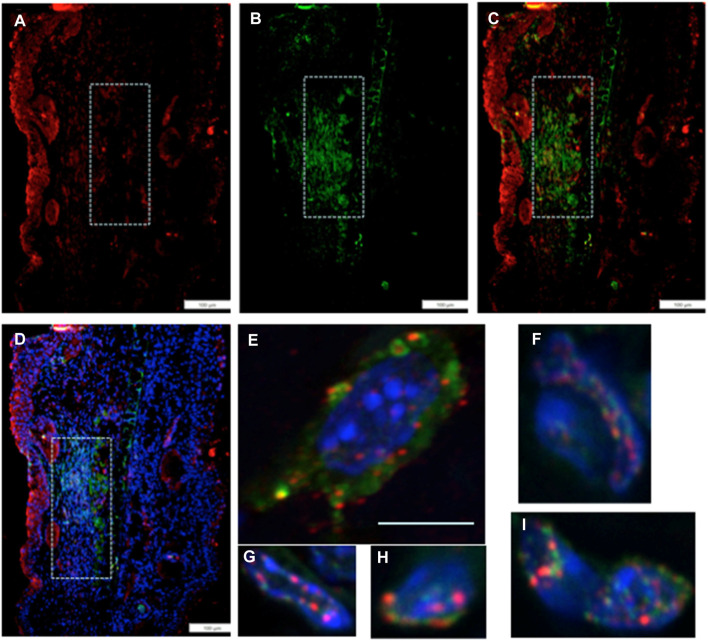
MRL ear tissue 7 days post-injury was analyzed by double-staining with an antibody for the EMT marker Twist1 (green) and for a marker of chromatin remodeling using an antibody specific for the PRC2 protein component EZH2 (red). The same area (white boxes) in the MRL blastema co-stained with an antibody to **(A)** EZH2 (red), **(B)** Twist1 (green), **(C)** a+b, and **(D)**a+b + DAPI again in the “EMT zone.” Confocal images **(E–I)** show EZH2 and Twist1 antibodies in single cells stained in the nucleus (blue, DAPI), nuclear membrane, and peri-nuclear region in the same region. Two blocks with ear holes from two different MRL mice and three consecutive sections per slide were double-stained. Data from one block are shown. Measuring bar = 100 microns. The measuring bar for confocal images = 5 microns.

We then investigated if the unusually high number of cells paused in G2M in regenerating tissue, which co-stained with an EMT marker, might also be engaged in chromatin remodeling. Thus, we examined the co-expression of Twist1, the EMT marker (green), with a chromatin remodeling marker EZH2 (red) (Figure 4). EZH2 shows staining in the same location as Twist1 (Figures 4A–D). Throughout that region, high-magnification confocal images show co-staining of individual cells for both markers, with EZH2 mainly associated with the nuclear membrane and Twist1 found in the perinuclear, cytoplasmic, and nuclear regions of the cells (Figures 4E–I).

### Co-expression of G2M and chromatin markers in individual cells

**FIGURE 5:**
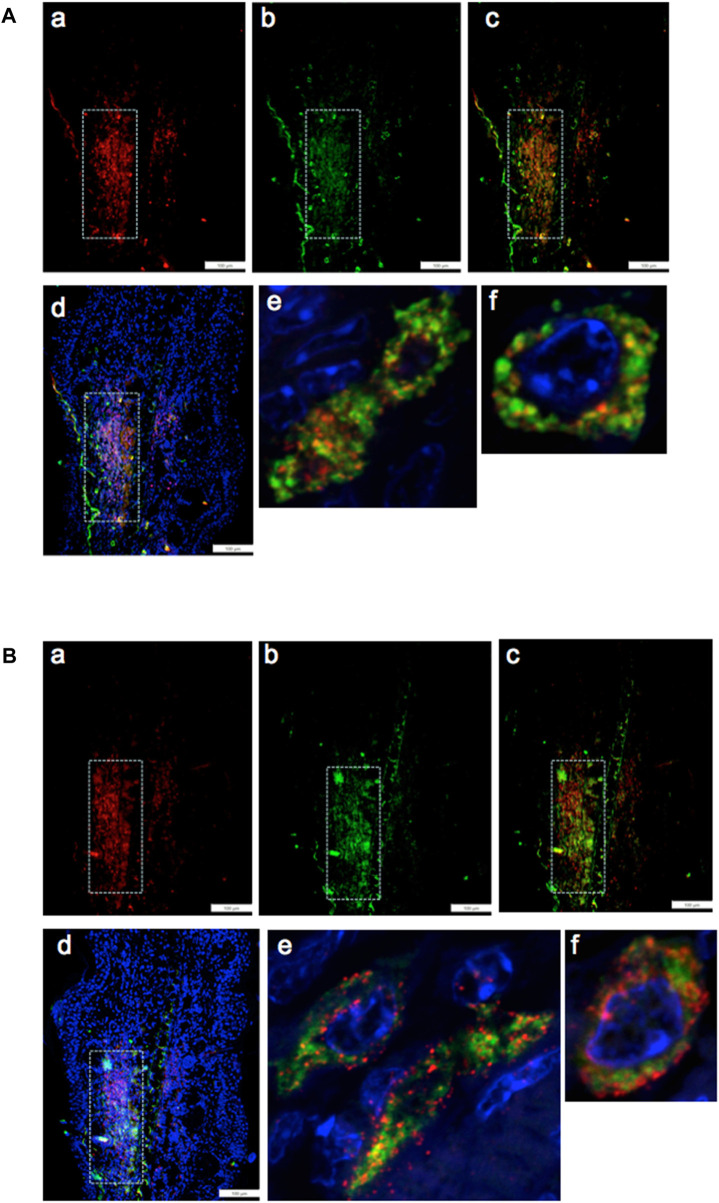
Ear tissue from MRL day 7 post-injury was analyzed pair-wise by double-staining with markers for G2M (EVI5) and the PRC2 component EZH2 **(A)** and double-staining with EVI5 and the chromatin marker H3K27me3 **(B)**, a product of EZH2 histone transmethylation. Tissue was co-stained with antibodies to a) EVI5 (red) and b) EZH2 (green) **(A)** or b) H3K27me3 (green) **(B)**, c) a+b, and d) a+b + DAPI (blue) to identify nuclei. Areas (white boxes) in the MRL blastema show overlapping staining in the “EMT zone.” Confocal images seen in **(A)** e,f and **(B)** e,f show same cell staining in the nucleus, nuclear membrane, and perinucleus of EVI5 and either EZH2 or H3K27me. Such single-cell staining was seen through the EMT zone. Two blocks with ear holes from two different MRL mice and three consecutive sections per slide were double-stained. Data from one block are shown. DAPI staining shows the nuclei. Measuring bar = 100 microns.

By pairwise analysis, we tested all possibilities of co-staining. We examined the G2 marker EVI-5 and its co-expression with the chromatin remodeling proteins, either EZH2 (Figure 5A) or H3K27me3 (Figure 5B), the histone H3 which is methylated by EZH2 on lys 27. Staining in both cases was observed in the same area, as observed with the previous antibodies (Figures 5A, B, a–d). Confocal imaging showed co-staining in single cells throughout the tissue and in the nucleus and the peri-nuclear region of the cell (Figures 5A, B, e).

### Pathway circuit analysis for all markers and the role of HIF-1α

**FIGURE 6:**
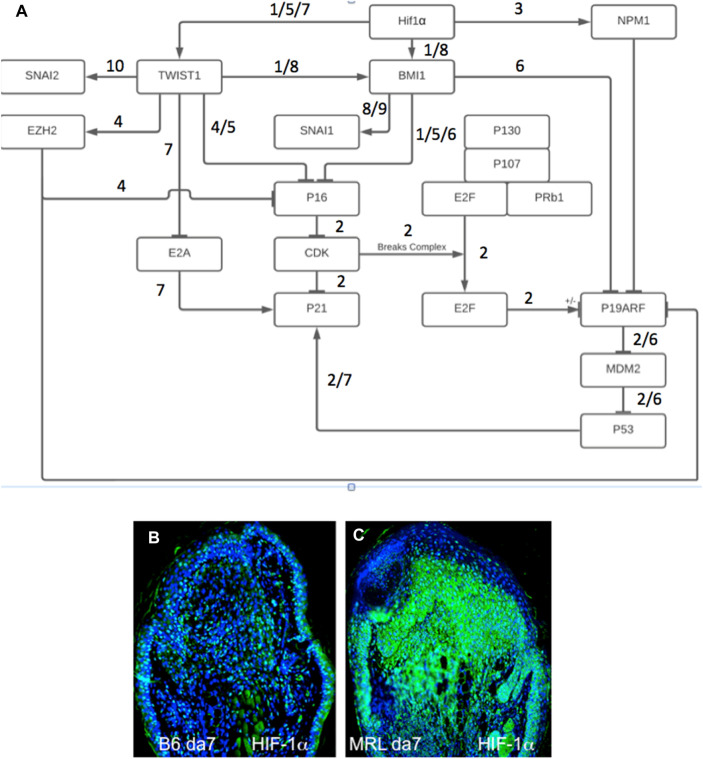
Gene circuit diagram for HIF regulation of EMT, chromatin remodeling, and cell cycle markers. This gene circuit is composed of canonical pathways (reference annotated), with HIF-1α as the initiator of downstream events reported herein **(A)**. HIF-1α has direct activation ties to EMT (Twist1) and chromatin remodeling (BMI-1, EZH2 through Twist1, and NPM1) and suppression of p19, p21, and p16 cell cycle checkpoint genes. The level of HIF-1α staining in MRL and B6 ear tissue on day 7 shows extensive staining throughout the MRL blastema **(B,C)**. Four blocks from four mice of each strain with three sections per slide were stained with an anti-HIF-1α antibody. Only one block from B6 and MRL is shown here. The numerically labeled pathways and gene nodes are referenced in Table 3. The measuring bar used in Figure 1= 50 microns applies to these photomicrographs.

We had previously shown the required upregulation of HIF-1α in regenerating MRL tissue with *siHIF1*α blocking regenerative ear hole closure (Zhang Y. et al., 2015). Figure 6A shows a gene circuit diagram showing the central role HIF-1α plays in EMT, chromatin remodeling response, and cell cycle control. Although HIF-1α is extensively expressed throughout the MRL ear blastema on day 7 post-punching injury (and not observed in the B6 ear) (Figures 6B, C), the other markers examined here and activated by HIF-1α show a very specifically defined area of expression. This supports the idea that HIF-1α expression does not turn on these genes throughout the whole blastemal region but, in fact, activates these genes very specifically and regionally (the EMT zone). The numerically labeled pathways are shown in Table 3.

**TABLE 3:** Gene Circuit References.

1.	Yang et al. (2010)
2.	Pomerantz and Blau (2013)
3.	Koukoulas et al. (2021)
4.	Cakouros et al. (2012)
5.	Yang and Wu (2008)
6.	Bhattacharya et al. (2015)
7.	Tsai et al. (2011)
8.	Du et al. (2014)
9.	Song et al. (2009)
10.	Lander et al. (2013)

### Temporal expression of HIF-1α/EMT/chromatin remodeling and G2M in injured ear tissue

**FIGURE 7:**
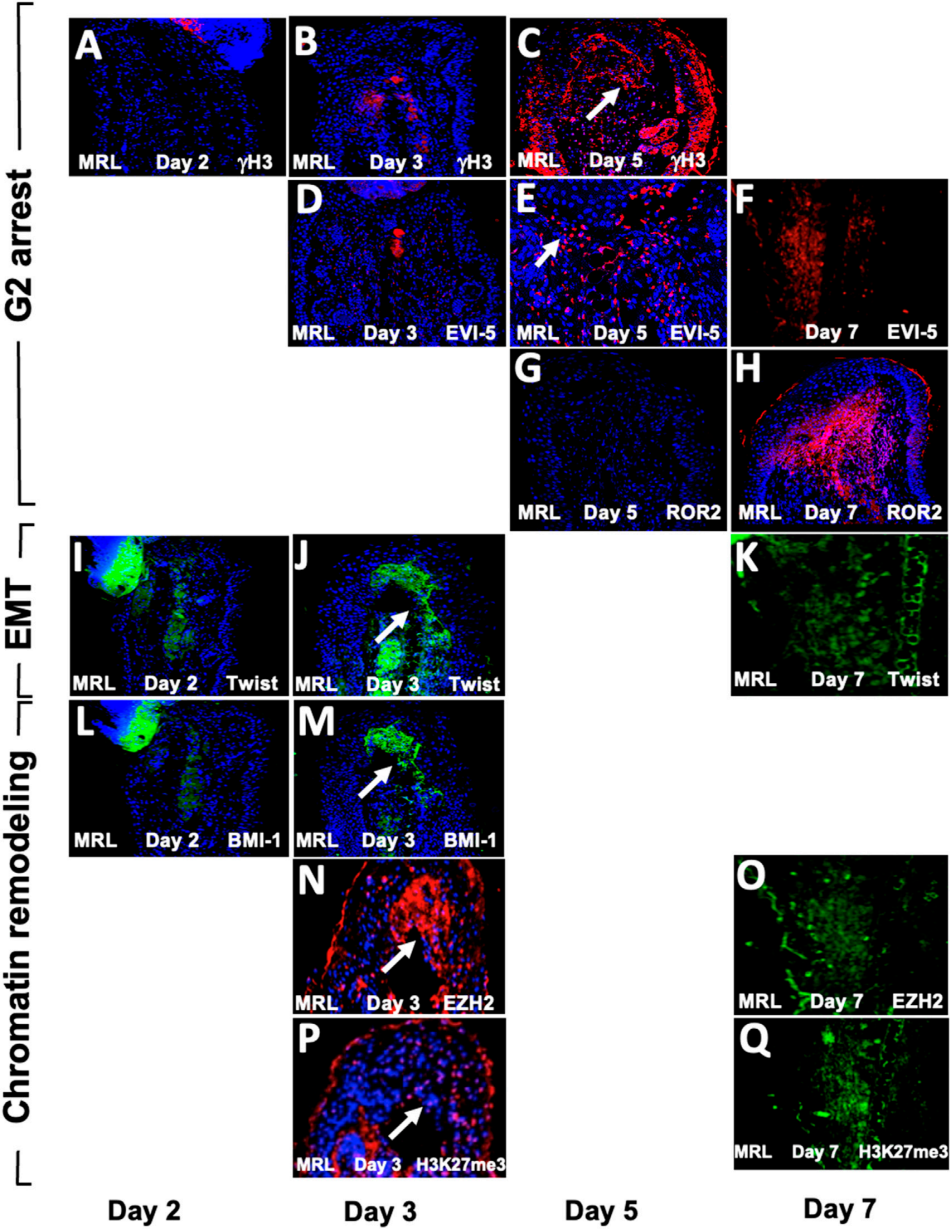
Temporal expression map of G2M, EMT, and chromatin remodeling functional histological markers. MRL ear tissue post-hole punch on days 2, 3, 5, and 7 has been stained with an antibody to multiple genes. IHC for G2 genes is given in **(A–H)**. γH3 and EVI-5 are expressed on day 5 but not day 3, and ROR2 is not positive on day 5 but positive on day 7. IHC for the EMT gene Twist **(I–K)** is positive on days 3 and 7 but not day 2. IHC for chromatin remodeling genes **(L–Q)** shows BMI-1, which is positive on day 3 but not day 2, and EZH2 and H3K27me3, which are both expressed on days 3 and 7. Areas of interest and IHC positivity are shown by a white arrowhead, and more highly magnified micrographs are seen for **(E)** (EVI5 da5) and **(P)** (H3K27me3, da3). The staining in **(P)** looks more diffused than in other positive figures. The staining seen in some of the micrographs, which were not considered positive **(A,B,D),** shows staining of the cartilage. Two blocks from two injured MRL mouse ears were used for each time point with three sections per slide. The scale bar in Figure 1, which equals 50 microns, applies to **(A–D)** and **(F–Q)**. **(E)** is magnified ×2.

Since the aforementioned results were obtained from day-7 post-injury tissue, it was of interest to determine the temporal expression of these markers to understand the sequence of events of these processes. MRL ear tissue was then stained between days 2 and 7 (Figure 7). The G2 markers EVI-5 and γH3 were expressed on day 5 but not on day 3 (Figures 7A–E), and ROR2 was expressed on day 7 but not on day 5 (Figures 7G, H). The EMT marker Twist1 was expressed on days 3 and 7 but not day 2 (Figures 7I–K). The chromatin remodeling PRC1 component BMI-1, and PRC2 component EZH2, and its histone target H3K27me3 were also expressed on days 3 and 7 but not day 2 (Figures 7L–Q). HIF-1α, which directly activates TWIST1 and BMI-1, was expressed early on day 0 and increased continuously, peaking at around days 7–10, as observed by IHC, bioluminescence, and Western blot analysis (Zhang Y. et al., 2015).

### FACS analysis shows markers for all three functional sets together in single cells

**FIGURE 8:**
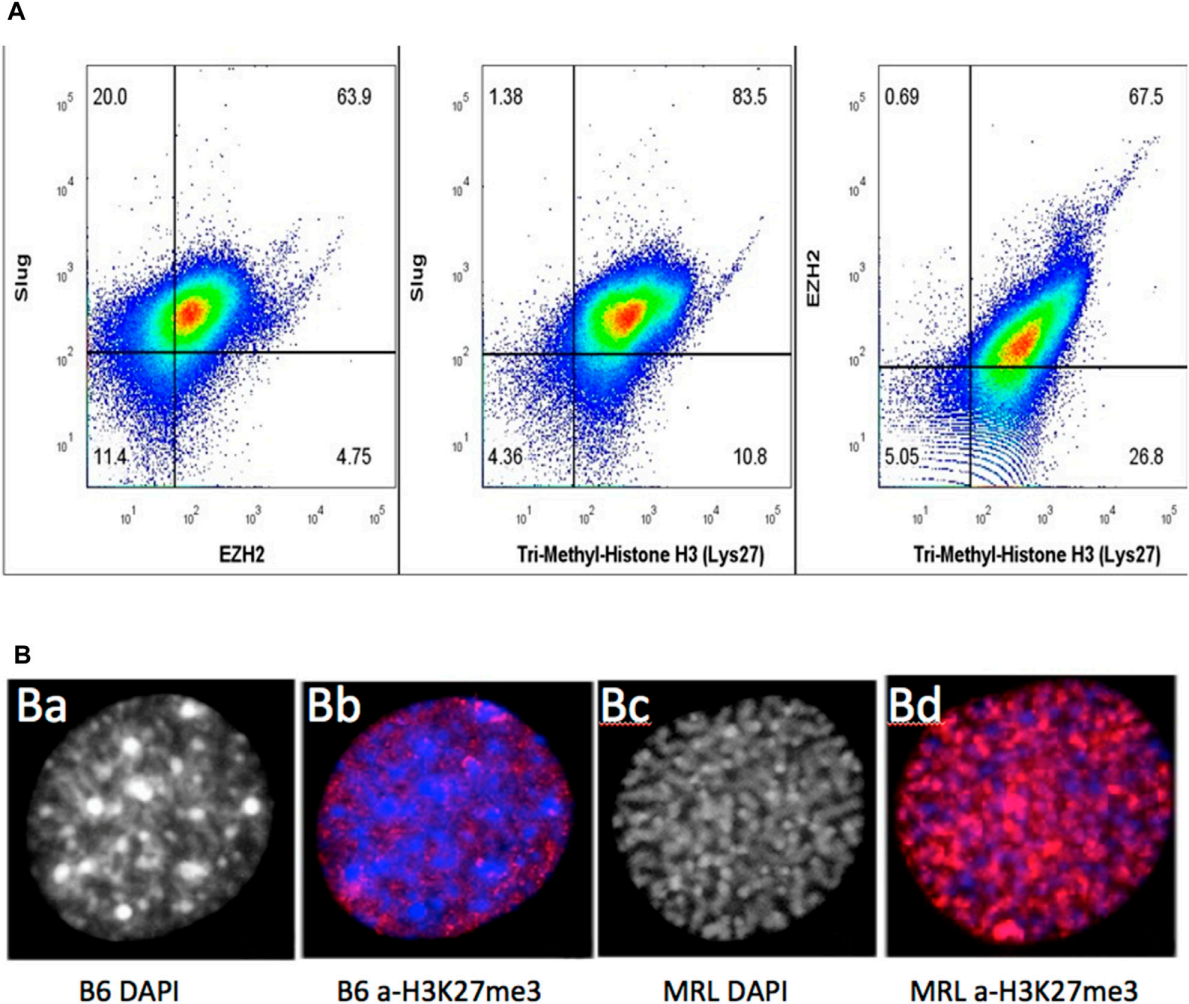
MRL ear tissue-derived cells show the overlap of G2, EMT, and chromatin remodeling markers. Uncultured MRL day 7 cells were stained intracellularly with directly labeled anti-Slug-APC, anti-EZH2-PE, and anti-H3K27me3 PE-Cy7 antibodies **(A)**. Attempts to label multiple anti-EVI5 antibodies showed no staining. Cells were then analyzed on BD Canto II and show overlap of the three markers in 79% of the cells (10,000 events) (Supplementary Figure S2). In a second experiment, to examine the intracellular localization of antibody binding specific to H3K27me3 (red), MRL and B6 fibroblasts were shown to be labeled only in the nucleus. B6 showed very faint staining with a condensed heterochromatin (prophase, DAPI) **(B)** (a), (**B)** (b) whereas MRL showed strong staining with an uncoiled euchromatin (G2, DAPI) **(B)** (c), **(B)** (d). DAPI staining in the MRL showed the relationship between G2M and H3K27me3 **(B)** (b,c). Approximately 100 cells for MRL and B6 each were analyzed and showed consistent staining, >85%, for each strain (see Supplementary Figure S3, field of MRL cells).

We initially examined MRL and B6 ear-derived fibroblast cells in culture, providing higher-resolution images of intracellular staining (Figure 8B). The majority of MRL cells were in the interphase (G2) (Figure 8B) and were stained for H3K27me3 (Figure 8B), combining G2M together with chromatin remodeling in a single cell and being highly expressed in the nucleus (Figure 8B). However, B6 cells showed little staining (Figure 8B).

Since the relationship between the cultured cells and the cells present in the ear tissue, which we know regenerates, is not determined, we then used cells derived directly from a day-7 MRL ear-punched pinna. Using three different antibodies against Slug (for EMT), EZH2 (chromatin remodeling), and H3K27me3 (chromatin remodeling), a pairwise analysis was carried out using intracellular FACS data on stained cells from ear tissue (Figure 8A). There was a significant part of the population (79%) that co-stained for Slug (EMT), EZH2, and H3K27me3 (chromatin remodeling). Unfortunately, an antibody for G2M (EVI-5 or γH3) to be used for FACS analysis could not be identified. However, with DAPI staining, over 85% of cultured MRL cell nuclei were shown to be in G2 (i.e., uncoiled chromosomes), as opposed to cultured B6 cells which showed condensed chromosomes (prophase) (Figure 8B; Supplementary Figure S3).

## Discussion

In our studies of mammalian regeneration in MRL/lpr and MRL/MpJ mice, complete scarless closure of a punched hole in the ear pinna occurs within 30 days with cellular and tissue events mirroring those observed in amphibian limb regeneration (Clark et al., 1998). This includes rapid re-epithelialization, observed in the amphibian within the first 12 h and in the MRL mouse within 24–48 h compared to the non-regenerator C57BL/6 mouse, which takes from 5 to 10 days (Clark et al., 1998). A second hallmark of amphibian regeneration, also observed early in the MRL regenerative response, is the breakdown of BM between the epidermis and dermis, permitting the cellular and molecular exchange of factors.

The reduction in blastemal mitosis observed not only in amphibians (Tassava et al., 1974; Mescher and Tassava, 1976) but also in hydra (Park et al., 1970; Buzgariu et al., 2018) and planaria (Salo and Baguna, 1984; Sahu et al., 2021) is a third hallmark of regeneration. This mitotic reduction and G2M arrest have features similar to those found in the MRL blastema and in both normal and blastemal cells in culture (Bedelbaeva et al., 2010; Heber-Katz et al., 2013). Cell cycle analysis revealed that the majority of these cultured cells were found to be in G2M, showed a DNA damage response expressing p53 and γH2AX, and lacked the expression of the p21^cip/waf^ protein (CDKN1a), a key G1 cell cycle checkpoint regulator. The lack of p21 in the regenerating MRL mouse predicted that its elimination in otherwise non-regenerating mice would convert these to regenerators. Indeed, p21 KO mouse ear holes could regenerate (close ear holes) like MRL (Bedelbaeva et al., 2010; Heber-Katz et al., 2013).

Until very recently (Sadler et al., 2019; Li et al., 2021), EMT, a core process in developmental biology, has received little attention in the context of regeneration with a notable exception (García-Arrarás et al., 2011). The overlapping localization of cells involved in the aforementioned processes supports the notion that EMT may be the source of functional cells in the blastema participating in de-differentiation, with in-migrating cells acting as bystanders or a supportive milieu. The observation of EMT concurrent with regeneration in organisms spanning evolution from echinoderms to mice suggests a deeper role for this developmental process.

Although the studies presented herein are strictly confined to the MRL and C57BL/6 strains of mice, which represent what occurs during regeneration (MRL) vs. what occurs during wound repair (C57BL/6), we made repeated comparisons to classical regenerating species including newts and axolotls, which are superior limb regenerators, and to echinoderm species such as sea cucumber, which displays the ability to completely regenerate its gut. Previous and ongoing studies in these species are a constant source of insight into mammalian studies.

### Regenerative epidermis

During the regenerative process, the epidermis plays a very special initiating role.

It receives injury signals; it covers the wound and does so rapidly. In the amphibian, it occurs within 12 h, and in the MRL mouse regenerating ear hole, it occurs within 24–48 h (Clark et al., 1998). It forms an apical epithelial cap (AEC), the epithelial structure that covers the wound but has a basal layer which expresses the mesenchymal marker fibronectin (FN) (Repesh et al., 1982; Nace and Tassava, 1995). In normal MRL mouse epidermis pre-wounding, Keratin 16, a gene associated with a keratinocyte activation state, is present at high levels and is not observed in the non-regenerating B6 epidermis (Cheng et al., 2013).

### EMT

Epithelial-to-mesenchymal transition is a process that is key to developmental events taking place in the embryo for neural crest formation, myogenesis including the heart, gastrulation, and stem cell trait acquisition and function, and tumorogenesis and metastases (Mani et al., 2008; Kalluri and Weinberg, 2009; Thiery et al., 2009; Kaowinn et al., 2017; Francou and Anderson, 2020). It has been reported to be important in regenerative processes in the sea cucumber and axolotl (García-Arrarás et al., 2011; Sadler et al., 2019). Although EMT is observed during wound repair, it is a very small and transient response within the first 24–72 h post-injury at least in the B6 mouse ear. In the regenerative process, however, as seen here in the MRL mouse ear, it is a full-blown response at least up to day 7. There are multiple stimulators of EMT, including hypoxia and molecular activation through molecules such as TGFβ and wnts (Mani et al., 2008).

In this process, epithelial cells, which normally express e-cadherin, cytokeratins, laminin, syndecan, claudin, and desmoplakin, are not motile. After the breakdown of the basement membrane in local tumor tissue, for example, they undergo changes by losing their polar epithelial characteristics, such as the expression of e-cadherin, and gain a migratory phenotype, acquiring fibroblast markers such as vimentin, fibronectin, FSP-1, Snail, Slug, Twist1, αSMA-1, FOXC2, ZEB1, and N-cadherin (Mani et al., 2008; Isert, 2023).

Changes in BM are due to MMP activation and remodeling, which is regulated by HIF-1α. In the amphibian, after limb amputation, BM does not reform between the epidermis and dermis due to continuous breakdown during the regenerative response. In the mammalian regenerative MRL mouse, MMPs are also activated by HIF-1α, and enzymatically active MMPs are observed as early as day 1 (Zhang Y. et al., 2015). BM breakdown can be viewed as the first permissive step in EMT because it is hard to imagine how any direct cell–cell contact or soluble factor diffusion can occur in the presence of an intact BM. *siHIF1a* blocks regeneration, MMP production, and BM breakdown. Conversely, upregulation of HIF-1α in a non-regenerative mouse leads to MMP activation, regeneration, and BM breakdown (Zhang Y. et al., 2015).

MRL BM, after injury, appears to reform on day 4 post-injury, remains intact until day 5, and then again disappears. It may be that on day 4, BM still allows cell crosstalk in MRL due to microbreaks in BM, as reported previously (Smit and Peeper, 2008). Thus, BM breakdown may happen before, during, and after EMT begins (EMT markers are upregulated on day 3) (Figures 2, 6) (Spaderna et al., 2006b; Walter et al., 2018). In the case of the B6 mouse strain, neither MMP expression nor BM breakdown is observed. There is an extensive literature on the breakdown of ECM as a permissive step to metastasis in cancer (Isert, 2023).

EMT is not only dependent on HIF-1α upstream (Chang et al., 2011; Zhang W. et al., 2015) but also on the molecule Twist1, a direct HIF-1α target (Smit and Peeper, 2008; Chang et al., 2011; Li et al., 2021), which turns off e-cadherin in mature epithelial cells. Interestingly, the molecule BMI-1 (a direct target of Twist1) is found in stem cells, resulting in the de-differentiation step which is so well-known in the formation of the blastema. BMI-1 is a component of the chromatin remodeling PRC1 complex and, thus, is permissive for chromatin remodeling to proceed. Knockdown of BMI-1 prevented changes induced by Twist and HIF-1α, leading to a lack of expression of stem cell markers (McCarthy, 2010; Yang et al., 2010).

### Chromatin remodeling

In addition to EMT driving chromatin remodeling, the reverse is also true (McCarthy, 2010; Yang et al., 2010; Bhattacharya et al., 2015; Sun and Fang, 2016). Four very different markers of chromatin remodeling are upregulated in the MRL blastema in the EMT zone: (i) the polycomb repressive complex 2 (PRC2) that contains transmethylase EZH2 and is involved in silencing gene expression by (ii) methylating histone H3 on its lysine 27 (H3K27me3), as shown by a specific antibody, and (iii) PRC1 binds to and blocks nucleosomes and limits transcription factor access using H3K27 to inhibit RNA pol2 initiation. This complex includes BMI-1, which is upregulated in the MRL ear on day 3. Lastly, (iv) nucleophosmin (NMP), a histone chaperone, is found in the nucleolus and binds to H2A and H2B (data not shown). Thus, EMT directly activates chromatin remodeling, and there are positive and negative feedback loops.

Not included in the data presented here is another molecule, HDAC3, involved in chromatin remodeling. This is a histone deactylase which has an enhancer activity affecting gene expression, is involved in modulating the chromatin structure in the nucleus, and is expressed on day 3 post-injury in MRL. Previous mapping of ear hole closure-associated genes showed that HDAC3 is a strong candidate and is upregulated in the MRL mouse (Cheverud et al., 2014).

### Temporal sequence

The day-7 IHC results of pairwise staining analysis, as well as the data on days 2, 3, and 5 (Figure 7), show that markers of all three processes are present throughout the timeframe of early blastema formation. These IHC studies also suggest the temporal order of EMT and chromatin remodeling as being the earliest events to occur, followed by G2. However, all three processes co-exist pairwise within the same cells, suggesting that all of the expression curves overlap to some degree. Interestingly, recent studies [(Luond et al., 2021), Luond, Develop Cell, 2021] have shown that EMT in metastatic breast cancer is highly variable with intermediate states and even partial reversal by MET. This is further complicated by the fact that there is also spatial variation within tumors. In the blastema, such variations may account for partial overlapping of IHC staining.

### *In vitro* studies

From histology, we observed that not all cells in any given region co-stain, and there are some regions of the ear blastema where no staining is observed. For *in vitro* studies, we isolated fibroblasts from normal unwounded ears from B6 and MRL mice cultured without further selection. The MRL cells showed a predominance of cells in G2M, unlike the B6 cells which were predominantly G0/G1, as shown previously (Michalopoulos, 2013). Here, FACS analysis of MRL fibroblasts showed co-straining with SLUG, an EMT marker, and EZH2, a chromatin remodeling marker.

However, better resolution could be observed with direct IHC of cultured cells, and the intracellular localization of molecular markers is readily apparent. Using an antibody to H3K27me3, we observed co-staining for DAPI, showing that the cells were in G2 and interphase, with the same cells expressing high levels of modified chromatin.

One might ask why these normal MRL cultured fibroblasts express markers not observed until after injury in the tissue. Given that the cells are isolated from tissue, are no longer affected by contact inhibition, removed from a normal *in vivo* cellular milieu, grown on plastic, and selected for growth over time, this might account for the early expression of markers.

### HIF-1α and metabolism

The upregulation of HIF-1α expression is an early marker of regeneration. The role of HIF-1α, in this regard, became clear because its expression in the regenerating MRL mouse was associated with the metabolic state used by the MRL mouse, which is more embryonic (Naviaux et al., 2009; Heber-Katz et al., 2015). The adult MRL employed aerobic glycolysis with increased lactate in preference to OXPHOS, a shift known to be regulated by HIF-1α. Another clue to the role of HIF-1α was the identification of RNF-7 (Sun and Li, 2013; Cheverud et al., 2014), part of the HIF-1α degradation pathway from MRL gene mapping studies. Furthermore, the drug dichloroacetic acid (DCA), a small-molecule inhibitor of PDK1, allows pyruvate to enter the mitochondria to produce ATP, shifting metabolism away from glycolysis and toward OXPHOS (Heber-Katz et al., 2015). This blockade of aerobic glycolysis by DCA also blocks regenerative healing in the MRL mouse. Confirmatory evidence for the critical role of HIF-1α was shown by the following: (a) blockage of HIF-1α using *siHif1*α leads to the blockage of regeneration and (b) upregulation of HIF-1α in otherwise non-regenerating mouse strains converts them to regenerators indistinguishable from the MRL (Zhang Y. et al., 2015). The latter was achieved using the PhD inhibitor, 1-4-DPCA, in a timed-release hydrogel formulation which downregulates the degradation of HIF-1α. In addition to ear hole closure, other regenerative models, such as enhanced and more rapid liver regeneration (Latona et al., 2017), and the complete recovery of a lost alveolar jawbone (Nagai et al., 2020; Zebrowitz et al., 2022) as a consequence of periodontal disease, are treatable with 1,4-DPCA. *SiHIF1*α completely blocked ear hole closure showing HIF-1α requirement in this response (Zhang Y. et al., 2015). Thus, HIF-1α is actually the central modulator of the genes examined here and is associated with EMT, G2M arrest, and chromatin remodeling.

A parallel relationship between metabolic reprogramming powering EMT in tumors is discussed by Jia et al. (2021). Here, TGFb is increased, which promotes glycolysis. The regenerating MRL mouse also shows increased TGFb expression (Kench et al., 1999).

### HIF-1α and cell cycle regulation

In the MRL regenerative response, we see that HIF-1α also directly activates Twist1 and EMT, BMI-1, EZH2 through Twist1, NPM-1, and chromatin remodeling. HIF-1α also indirectly suppresses p19, p21, p16, and cell cycle checkpoints. This answers our original question as to why there is so little mitosis in the blastema, and it also ties together important events during regeneration, EMT, chromatin remodeling, and cell cycle regulation. However, why is G2 arrest so critical to regeneration, as seen by the fact that the p21 KO mouse recreates the MRL regenerative phenotype? A possible answer is that p21 KO, in fact, upregulates HIF-1α. One interesting finding is that lincRNA-p21 inhibits HIF-1α and when lincRNA-p21 is off, HIF-1α is up (Ye et al., 2019). This requires further exploration. From our temporal IHC results, we show that EMT and chromatin remodeling occur at approximately the same time, followed by G2 arrest. There are developmental studies suggesting this order, such as EMT (Lovisa et al., 2015), leading to chromatin remodeling, which leads to cell cycle changes (Krebs et al., 2000; Ma et al., 2015). Reversing chromatin remodeling through the phosphorylation of EZH2 with the resultant inhibition of H3K27 methylation by the cell cycle checkpoint kinase CDK1, which acts in G2 and induces mitosis, leads to differentiation of stem-like cells, providing a possible mechanism for ending the blastema phase of regeneration (Wei et al., 2011).

In tumors, a novel finding showed that wtp53 binding to MDM and SLUG led to SLUG degradation and lack of EMT, whereas the mutant p53 led to undegraded SLUG and successful EMT (Wang et al., 2009). However, the MRL mouse, which is tumor-resistant and suggests wtp53 (Heber-Katz et al., 2015), and p53wt mouse strains treated with 1,4-DPCA (Zhang Y. et al., 2015) show both regeneration and ongoing EMT.

### HIF-1α and spatial effects

A very interesting observation arose early in our MRL HIF-1α studies. Using a HIF-1α-luciferase reporter MRL mouse and simply ear-punching, a minor localized wound, HIF-1α expression was observed in full body—over 90% of the animal glowed—with an IVIS SCAN (Zhang Y. et al., 2015). Thus, while the HIF-1α response was pan-tissue, the regeneration-competent cells were apparently localized to the wound site and may be only within a portion of the wound. In the current study, HIF-1α expression in the 7-day ear is clearly beyond the EMT zone, suggesting that not all cells are regeneration-competent in the ear, even though HIF-1α is being expressed in those cells.

## Conclusion

The striking similarity of biological processes that occur at the site of a wound in regeneration-competent species across phyla suggests an evolutionarily conserved organizing principle. A close dissection of the MRL mouse blastema using established molecular markers and their temporal order of appearance identify the epithelial–mesenchymal transition process, following HIF-1α expression as a strong candidate for this organizing principle.

## Data Availability

The original contributions presented in the study are included in the article/Supplementary Material; further inquiries can be directed to the corresponding author.

## Supplementary material

SUPPLEMENTARY FIGURE S1 Fibroblasts from MRL ears showed high accumulation in G2M. Cells from C57BL/6 ear pinnae **(A)** and MRL ear pinnae **(B)** were examined by propidium iodide staining and FACS analysis to determine cells in different stages of the cell cycle. Immunohistochemical staining of MRL ear tissue on day 7 post-injury shows molecules associated with G2M. These images were overlaid onto H&E sections from the same block. Images for Wnt5a are given in **(C,D)** and for ROR2 are given in **(D–F)**. **(A,B)** were reproduced from Bedebaeva et al. 2010, PNAS.

SUPPLEMENTARY FIGURE S2 Triple staining of cells in the day-7 MRL blastema. On the left is a histogram overlay of H3K27me3 expression for all four populations (EZH2+Slug+, EZH+Slug−, EZH2-Slug+, and EZH2-Slug−). On the right is a bar graph of the median fluorescent intensity (MFI) of H3K27me3 from each histogram on the left. The highest expression of H3K27me3 is in the EZH2+Slug+ population.

SUPPLEMENTARY FIGURE S3 MRL-derived fibroblasts stained with an antibody to H3K27me3. Seen above is a field of MRL-derived fibroblasts fixed on coverslips, stained with anti-H3K27me3, and counted. A single cell from this population is shown in Figure 8B. Over 100 cells were counted, and intensely stained cells were found to be >85% positive in this population.

## Abbreviations

BMI-1, B-cell-specific Moloney murine leukemia virus integration site 1, PRC1 component; EMT, epithelial-to-mesenchymal transition; EVI-5, ecotropic viral integration site 5 protein homolog; EZH2, enhancer of zeste homolog 2; IHC, immunohistochemistry, PRC2 component; H3K27me3, methylated histone 3 lysine 27; HIF-1α, hypoxia-inducible factor 1α; PRC, polycomb repressive complex.
