# Supplementary figures and images for: Epithelial–mesenchymal transition: an organizing principle of mammalian regeneration
Source: Front Cell Dev Biol. 2023 Oct 26;11:1101480. doi: 10.3389/fcell.2023.1101480 (PMC10641390; doi:10.3389/fcell.2023.1101480)

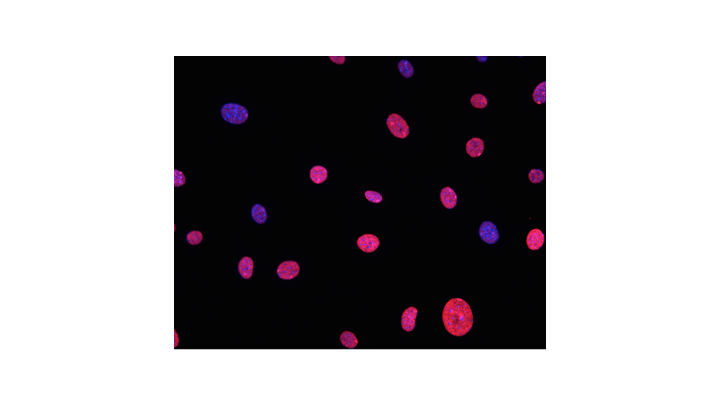

Supplement: Supplementary file 1 [file Image3.tiff]

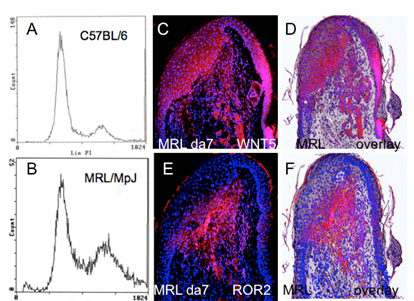

Supplement: Supplementary file 2 [file Image1.TIF]

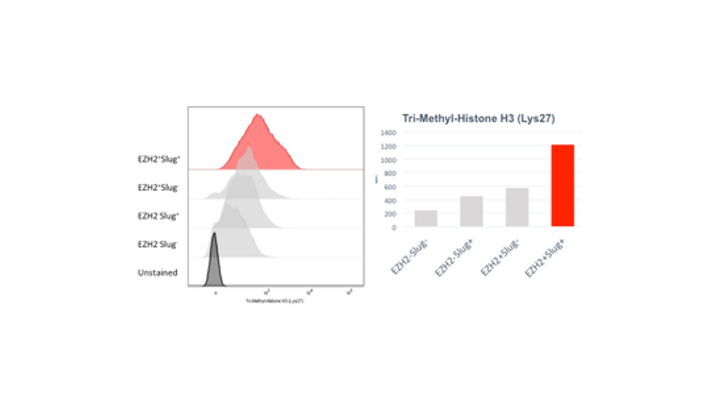

Supplement: Supplementary file 3 [file Image2.tiff]
